# Starting university during the pandemic: First-year international students’ complex transitions under online and hybrid-learning conditions

**DOI:** 10.3389/fpsyg.2023.1111332

**Published:** 2023-02-16

**Authors:** Lizhou Wang

**Affiliations:** Center for International Higher Education, Boston College, Chestnut Hill, MA, United States

**Keywords:** international education, higher education, college transition, online education, international student, COVID-19, transition theory, hybrid learning

## Abstract

International education activities suddenly halted due to the COVID-19 pandemic, severely disrupting student mobility and academic learning. Many educational institutions have delivered programs to students through digital devices globally rather than *in situ*. Such a shift presents a unique opportunity to assess the impact of online and hybrid education for international students. This qualitative study interviewed 30 international students who had arrived on campus and shared their first-year university transition experiences during the pandemic. The analysis shows that spatial and temporal circumstances created two scenarios and thus resulted in different first-year university experiences. Although all students were dissatisfied with online learning, studying at a distance in different time zones was particularly detrimental to international students’ mental and physical health. The (im)mobile environments led to mismatches in expectations, roles, activities, and realities, negatively affecting student learning and adjustment. This study highlights the complex international transition issues and offers implications for sustainable online and hybrid-learning practices in the educational system.

## 1. Introduction

COVID-19 has caused a historic disruption in education, impacting more than 220 million tertiary-level students worldwide (UNESCO, 2021). International students are among the most affected group: before international education activities suddenly halted, over 5.6 million higher education students studied internationally in 2019–2020 (IIE, 2020). All major international student host countries, including the United States, United Kingdom, Canada, Australia, Japan, and China, had imposed strict border control policies and travel restrictions that prevented international student entrance and international mobility (Buckner et al., 2022). According to the International Association of Universities, 89% of higher education institutions that responded to The Global Survey reported that COVID-19 impacted international student mobility (Marinoni et al., 2020). Consequently, educational institutions have delivered programs through digital devices to students across the globe rather than *in situ*, suggesting that different forms of online education could provide new opportunities and directions for the internationalization of tertiary institutions (de Wit and Altbach, 2020).

Meanwhile, studies have emerged to empirically examine the precarious conditions that international students faced due to international travel restrictions and online learning. The global pandemic has imposed additional challenges to traditional language and cultural adjustment barriers because international students could no longer travel to campus. The on-campus residential experiences have long been found to offer meaningful and transformational education that fosters college student development (Schlossberg, 1981; Bronfenbrenner, 1994; Astin, 1999; Patton et al., 2016). Thus, the pandemic-related changes to international student transitions raise questions about transnational higher education experiences.

This study presents the findings from 30 in-depth interviews with university students who reflected upon their first-year transition during the pandemic, responding to calls for a comprehensive empirical analysis of diverse student experiences in transnational higher education (King and Raghuram, 2013). The participants are a sub-group of international students, those who graduated from international high schools and exhibited several distinctive characteristics worth researching. Firstly, like all first-year university students, they are in a critical developmental stage, and going to college is an essential step toward independence and adulthood. Secondly, having attended international high schools with English as the medium of instruction, these students may perceive fewer cultural and linguistic obstacles. As such, their challenges could not be simplified or limited to cultural, linguistic, or pedagogical mis-adjustment. So, this group of students offers a “unique opportunity” to assess the difficulties and problems in international university transition during the pandemic. Thirdly, this study adds evidence to the under-researched but fast-growing population seeking oversea degrees: over 12,000 international schools taught more than 6 million students in 2021 (ISC Research, 2021).

This research applies the theoretical transition model within the person-environment framework to understand the stories of first-year international students who persisted through “multiple transitions” in their first year of university under unexpected situations. The time and place factors are salient in differentiating the experiences of hybrid-learning and fully online students. This study contributes to the scholarly conversation on how the learning conditions impact young people’s physical and mental health and offers implications for future online education and virtual mobility.

## 2. Research questions

This qualitative paper used a phenomenological approach to understand the experiences of transitioning into university for international students during the pandemic. The overarching research question is: How did international students perceive their first-year university transitions during the global pandemic? More specifically, how did students perceive online and hybrid learning? What strategies did students perceive as effective during challenging times?

### 2.1. Definitions of terms

#### 2.1.1. International students

As modes of delivery, content, and providers in international higher education have become increasingly international, a broad definition tends to neglect the complexity of international student mobility (de Wit, 2008; Wang L., 2022). The commonly used definitions by UNESCO and OECD emphasize border-crossing and visa-issuing, yet both activities were paused during the pandemic. Instead, this study stresses the *international* component of the concept, so *international students* refer to degree-seeking students who attend universities in a country different from their passport countries or high school countries.

#### 2.1.2. International schools

K-12 international schools take many forms. The characteristic that all international schools share is that the curriculum is not of the home country (Hayden and Thompson, 2013). In the current study, the participants graduated from international schools in Asia. These international schools are accredited by the Council of International Schools, the high school curriculum is the academically rigorous International Baccalaureate Diploma Program, and the language of instruction is English. These schools primarily serve international mobile families and the local higher socioeconomic status families.

#### 2.1.3. Hybrid learning versus fully online mode

All students had to learn online and take classes *via* Zoom during the pandemic, but study participants experienced two forms of online learning. Hybrid-learning mode refers to a condition that, in addition to learning online, students experienced in-person interactions, whether it is sitting in classrooms or accessing on-campus facilities. For study participants who were able to enter the university country during the pandemic, they lived close to campus and belonged to this category. Fully online mode means students were physically located in their home countries. They spent an academic semester or a year online and participated in synchronous and asynchronous classes.

#### 2.1.4. Higher education institutions, colleges, and universities

These terms are used interchangeably throughout the study to refer to the same post-secondary degree-granting stage. Because the study participants are in different countries, they have different colloquial and cultural preferences in naming this stage.

#### 2.1.5. Transitions

*Transitions* in this research describe the process of students entering universities and adjusting to the new environment. Participants were interviewed after completing at least 1 year of university. The theoretical framework section discusses the transition model in more detail.

## 3. Literature review

Extensive literature on international students in higher education has highlighted their challenges and obstacles. The most studied themes are cross-cultural adjustment (transition/acculturation/adaptation), mental health problems (stress/depression), second-language acquisition, intercultural development (competency), and student migration (labor market/push-pull factors; Jing et al., 2020). For degree-pursuing students, literature has primarily focused on socio-cultural, academic, emotional well-being, and adjustment issues (Gümüş et al., 2020). Critical factors influencing international student adjustment include early life experiences, resilience, self-efficacy, spiritual and social support, coping style, personality, and emotional and cultural intelligence (Mesidor and Sly, 2016).

Due to the pandemic, international and domestic travel restrictions, socio-political events, communal hate crimes, and financial impacts (relating to scholarships, tuition fees, and incomes) have put international students in precarious positions, particularly in the leading host nations (Anandavalli et al., 2020; Alaklabi et al., 2021; Buckner et al., 2022). Safety, health, mental health, and racial biases have adversely affected students of color, Chinese students, and Asian students in the United States and Canada (Blake et al., 2021; Ge, 2021; Gao et al., 2022).

Examining international students currently residing in the host countries, many researchers proposed ways for institutions to provide adequate psycho-social support. Large-scale quantitative assessments have identified that international students were under social, emotional, academic, and discrimination stresses (Soria and Horgos, 2021; Gao et al., 2022). Many qualitative research further pinpoint that international students face limited opportunities to interact with family, peers, and faculty members and therefore need to secure transnational support (Wang and DeLaquil, 2020; Han et al., 2021; Hari et al., 2021; Hu et al., 2022).

While the above studies focused on the students already on campus, less research has explored the experiences and infrastructure support of non-traveling international students. Wang B. (2022) analyzed the case of 23 Chinese international graduate students and showed how they coped with asynchronous learning through self-agency and flexible institutional arrangements. These graduate students demonstrated a high agency level to mitigate the adverse immobility conditions. From an institutional support angle, Mann and Mennicke (2022) detailed how Franklin and Marshall College in the US designed a special program for first-year Chinese students physically separated from the college campus. According to both studies, synchronous and blended learning sessions were effective techniques for supporting students who remained in their home countries.

The student population who transitioned from Asian international schools to Anglophone higher education has been growing yet received little attention. One related research study examines International Baccalaureate Diploma Program graduates in higher education. In these studies, students’ high schools are in anglophone countries (the United Kingdom, the United States, Canada, and Australia), and students tend to continue to attend higher education institutions within these contexts. As such, the students encountered fewer transitional difficulties because they were in the same countries. Research demonstrates that there is high pressure and stress associated with the International Baccalaureate high school curriculum, and students report being well-prepared for college in terms of entrance, retention, and competition (Coates et al., 2007; Hertberg-Davis and Callahan, 2008; Culross and Tarver, 2011; Perna et al., 2015; Suldo et al., 2018). Recent research in non-Western contexts has also begun concentrating on International Baccalaureate graduates in universities. The interviewees acknowledged that to have gained important skills pertaining to higher education learning but encountered challenges due to unfamiliarity with the “eastern” pedagogy and assessment in Hong Kong and South Korean universities (Wright and Lee 2020; Park and Hong 2021). It remains to be discovered whether students from Asian international high schools transitioning into western contexts would face similar issues.

Another connected research on international school alum focuses on their identities and flexibility, given various changes. Previous studies used the concepts of *Global Nomads* or *Third Culture Kids (TCKs)* to describe students who have spent significant parts of their developmental years outside their parents’ culture(s) (Hayden, 2006; Pollock and Van Reken, 2006; Grimshaw and Sears, 2008). With increased globalization and mobility, these terms are used interchangeably to describe internationally mobile children (Langford, 2012). TCKs are privileged materially, socially (in terms of social and communication skills developed), and educationally through first-hand experiences of history, geography, religion, languages, and cultures that other children might learn about only through books or the Internet (Hayden, 2006). As such, self-identifying as a TCK may indicate that the student is perhaps more prepared for unexpected situations and international transitions.

Collectively, the literature supports the need to understand how international high school graduates transitioned into university during the pandemic. Empirical research on the transition to online teaching and learning in higher education has been limited, and several gaps exist. One example is the predicament facing first-year students: they were new to a university, had limited exposure to the surroundings, and lacked facilities comparable to those provided by Franklin and Marshall College. It begs the theoretical assessment of the transitions among students. Moreover, more research is needed to discover whether students whose language of instruction in high school is English will also have adjustment difficulties while entering western anglophone higher education institutions. The pandemic put students in a dire situation, which motivated an empirical examination of such impacts.

## 4. Theoretical framework

Theories from the person-environment framework guided the research design, data collection, and analysis. To best understand how students experienced transitions during the pandemic, two theories within the person-environment framework: ecological systems theory and transition theory, are utilized (Schlossberg, 1981; Bronfenbrenner, 1994; Goodman et al., 2006). The person-environment framework serves as a lens for analyzing college student growth and outcome, and embedding transitions within the ecological systems provides a way to understand and assess student development holistically (Ballo et al., 2019). It incorporates the interdependent variables of ideology and culture, social and organizational structure, time, and individual agency (Arnold et al., 2012).

A college student’s development is nested within and affected by relationships in the environmental systems: from the immediate face-to-face settings to the most distal broader social contexts (Bronfenbrenner, 1994). In Bronfenbrenner’s terms, there are five systems in this model: (1) microsystem is the student’s immediate environment (e.g., family, friends); (2) mesosystem is the connections within the immediate environment; (3) exosystem is the external settings that indirectly affect development; (4) macrosystem is the larger cultural and social context; and (5) chronosystem encompasses the patterns and transitions over a particular time. Notably, COVID-19 is a macrosystem and time-specific event, which invariably affects students’ experiences from the microsystem to the macrosystem. The intersection of individual life and sociohistorical context is a crucial determinant of student development because they are “shaped in part by the era in which they attend college” (Renn and Arnold, 2003, p. 272).

The transition model consists of three process stages: “moving in,” where the individual student tries to figure out the new environment, “moving through,” as the individual adjusts to the new environment, and “moving out,” as the individual leaves the environment (Anderson et al., 2011). This study focuses on the “moving in and moving through” stages, as international students attended universities and tried to adjust to the new settings.

**Figure 1:**
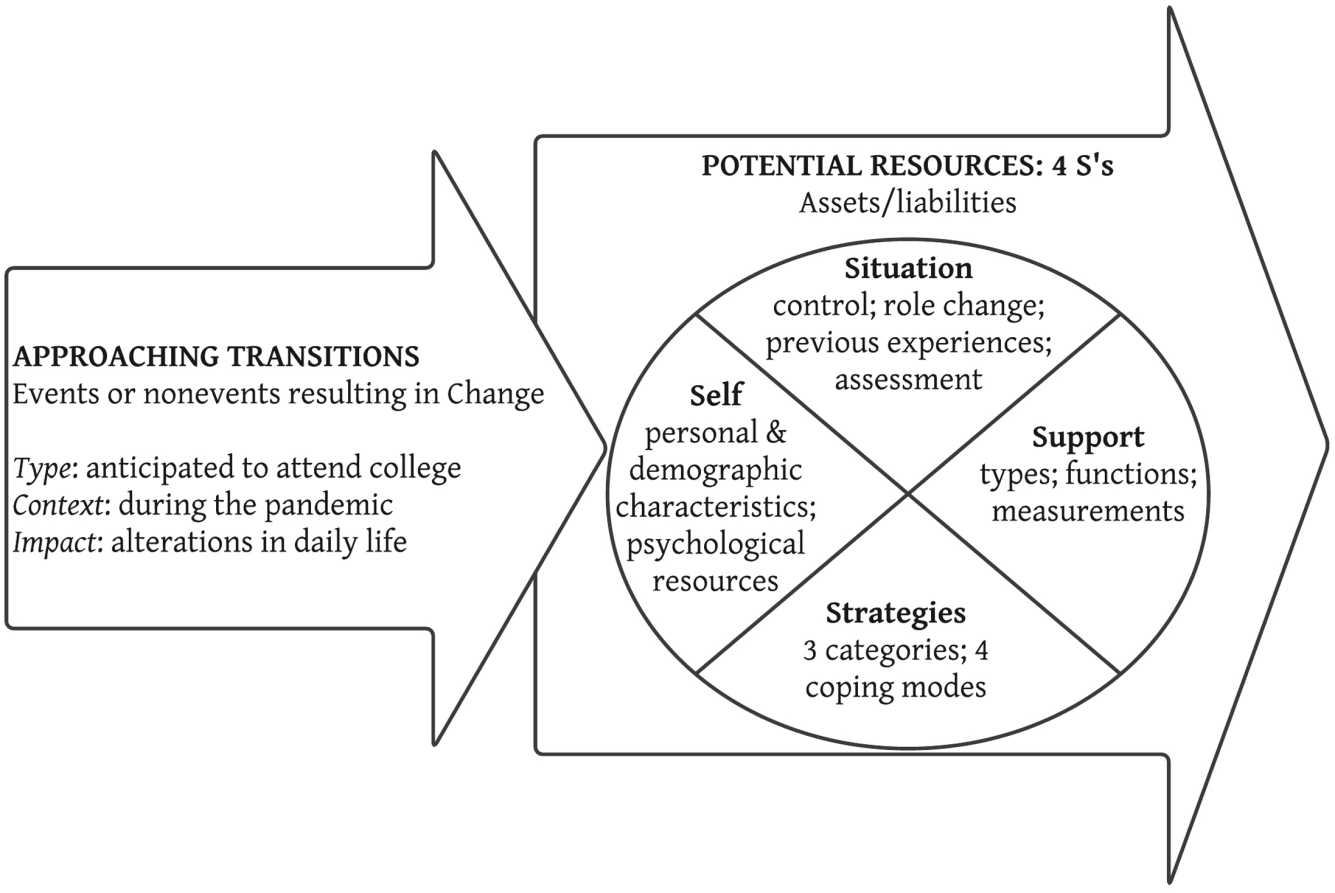
The transition process: moving in and moving through.

Transitions produce stress and require coping, with “four main factors [impacting] an individual’s abilities to deal with a transition” (Patton et al., 2016, p. 48). Figure 1 shows the transnational university transition model during the pandemic^1^, the four main factors that influence student transition are termed the 4 S’s: *Situation, Self, Support, and Strategy*; these are the resources that an individual student possesses during the transition. This framework suggests that going to university was a *voluntary anticipated event* that these students planned for over the past years. The *context* of the global pandemic would affect their reactions, which has an *impact* on their daily lives. Using this frame allows questions to be designed and asked on students’ perceptions on the situation, self, support, and strategy.

## 5. Methods

### 5.1. Research design

The entire research project began in the summer of 2021 and concluded in the summer of 2022. It was designed to be a mixed-method project to explore students’ transitions from international high schools to higher education during the global pandemic, combining cross-sectional surveys and phenomenological interviews. The survey assessed how students’ overall remote college experiences were affected by factors at various life stages using UCLA’s Freshmen Survey 2021 edition (Higher Education Research Institute, 2021). Using a phenomenological approach, this paper specifically reports the findings from in-depth interviews to explore and explain the international transitioning experiences (Creswell and Poth, 2017). Semi-structured interview questions covered identities, personalities, aspirations, family influences, high school curriculum and environment, pre-college expectations, college choices, academics, social life, and daily routine. Interviews took place in the summer of 2021 and 2022 after students had completed their first year of university education.

### 5.2. Research participants

The research population comprises students who fulfill the following criteria: graduated from international high schools in Asia; completed their first year of university in a country different from their passport country or high school country. As such, the participants could effectively reflect upon their transnational transition experiences as first-year university students from 2020 to 2021. Research participants were recruited through purposive and snowball sampling (Merriam and Tisdell, 2015). This research was promoted by the Council of International Schools and the International Baccalaureate within their networks. A few school directors and principals in Asia responded to the call and sent emails to reach their alum base. Participants also recommended that their friends and classmates join the interview. Thirty interviewees participated in this study: 18 were fully online, and 12 were hybrid-learning students. They came from small-sized international high schools in Asia (China, Hong Kong SAR, India, Indonesia, Malaysia, Singapore, and Vietnam) that graduated 21 to 150 students per academic year.

By design, the participants in this study cohort share many features with other Asian international high school students (Fraenkel et al., 2019). All of them demonstrated an exceptional command of the English language. Seventeen students self-identify as Third Culture Kids; many grew up and attended high schools outside of their passport countries. All interviewees are enrolled in world-class universities or highly selective liberal arts schools in Australia, the United States, the United Kingdom, Canada, and English-medium-instruction institutions in Europe, Hong Kong, Japan, and Malaysia. Many could not enter the host country due to international or host-nation-imposed travel restrictions. All have many previous international travel experiences, including moving to, attending, or visiting college campuses in other countries. Their families support their academic endeavors and pay for their tuition. Thus, although that half of the students held part-time jobs, they did not experience substantial financial stress at this time. After the first year of university, most students were overall satisfied with their academic achievements and accomplishments. Table 1 presents detailed information about each interview participant.^2^

**Table 1:** Interviewee demographics.

Code	Gender	High school country	Passport country	Third cultural kid	University location	Major
*Fully online students*						
ST1	Male	China	Hong Kong	No	USA	Biochemistry and religion
ST2	Female	China	Hong Kong	No	USA	Literature and history
ST3	Female	China	Australia	Yes	UK	Business
ST4	Female	India	India	No	USA	Psychology
ST5	Female	Thailand	Thailand	No	USA	Chemical engineering and biology
ST6	Male	Thailand	USA	No	USA	Political science
ST7	Female	China	Australia	No	USA	Philosophy and economics
ST8	Female	India	India	Yes	Canada	Psychology
ST9	Male	Thailand	Myanmar	Yes	USA	Economics
ST10	Female	Thailand	Thailand	No	Canada	Business
ST11	Male	Malaysia	Vietnam	Yes	USA	Mechanical engineering
ST12	Female	Indonesia	Indonesia	No	UK	Biomedical science
ST13	Female	Thailand	Thailand	No	USA	Biochemistry
ST14	Female	Vietnam	Vietnam	No	Australia	Pharmacy
ST15	Male	Mongolia	Mongolia	No	Japan	Digital business
ST16	Male	Malaysia	Malaysia	Yes	UK	Political science
ST17	Male	Thailand	Myanmar	Yes	USA	International relations and economics
ST18	Female	India	India	Yes	Canada	Psychology
*Hybrid-learning students*						
ST19	Female	China	Spain	Yes	UK	Genetics
ST20	Female	Hong Kong	The Philippines	Yes	Europe	Music vocal performance
ST21	Female	Hong Kong	Canada	Yes	USA	Arts
ST22	Male	Thailand	USA	Yes	USA	Communications
ST23	Female	Hong Kong	Nepal	Yes	Hong Kong	medical science
ST24	Female	Singapore	India	Yes	UK	Medical science
ST25	Female	Hong Kong	Nepal	Yes	Hong Kong	Applied medical science
ST26	Male	Indonesia	Indonesia	No	USA	Economics and theology
ST27	Male	Malaysia	Pakistan	Yes	Malaysia	Computer science
ST28	Male	Singapore	India	Yes	Canada	Aerospace engineering
ST29	Male	Malaysia	Vietnam	Yes	USA	Engineering
ST30	Female	China	USA	Yes	USA	International relations

### 5.3. Data collection and analysis

Data collection began after obtaining Institutional Review Board approval from the researcher’s institution’s Office for Research Protections. After signing the consent forms, 10 students joined the individual interviews in the summer of 2021. A year later, in the summer of 2022, 30 students participated in the interviews. Among them, eight were participants from 2021 who decided to join for the second round of the interview. The data became robust with the longitudinal component, as these interviews substantiated and verified the findings. Because of the travel restrictions still imposed by different governments, all interviews were conducted online *via* Zoom. Interviews were conducted in English, recorded with consent, and transcribed verbatim. Each interview ranged from 60 min to 90 min.

### 5.4. Data analysis

The interview analysis used a deductive method to answer the research questions within the theoretical model. Interview data were coded and analyzed with two rounds of memo writing and coding in NVivo 12 and then MAXQDA, allowing chances for re-examination, synthesizing, and merging of the codes in additional cycles (Erickson, 2012; Saldaña, 2015). The first round of coding generation and analysis began with an analytic memo writing session after each interview (Merriam and Tisdell, 2015). These memos captured observations, reflections, and ideas for possible initial codes. Codes were refined iteratively to identify key themes in discussions and presentations with research center members.

Because the transition model stresses students’ evaluation and appraisal of the events, affective coding methods were applied to investigate the subjective qualities of participants’ experiences, which are “core motives for human action, reaction, and interaction” (Saldaña, 2015, p. 124). These codes include emotions (confusion/relief/doubt), values (attitudes/belief/motivation), versus (us/them, professor/me), and, very importantly, appraisal and evaluations (positive/negative).

## 6. Results

### 6.1. Overall negative assessment of online learning

The 30 in-depth interviews provided explanations for the survey findings, which identified an overall unfavorable to a neutral appraisal of students’ college experiences in hybrid and online contexts. In the survey, students evaluated programs relating to *orientation*, *first-year integration*, and *counseling and well-being support* as “not very satisfied.” In the interviews, students viewed their first year as crucial for establishing a new identity, forming new social ties, exploring *via* trial and error, and preparing for more advanced study. The contrast indicates the inadequate formation of institutional support in the students’ exosystems, which, in turn, affected the structure of their mesosystem and microsystem.

Regarding academic learning, the *effectiveness of remote instruction* received the lowest rating in the survey. However, interviewees clarified that it was not about professors’ or lecturers’ teaching and pedagogical styles. Instead, they interpreted the ineffectiveness as a result of three interrelated factors: *lack of engagement and contact, fatigue, and distraction*. All student interviewees stressed the issues of Zoom and online fatigue and boredom. Such issues caused behaviors relating to procrastination and distraction after a lengthy period spent online. Students frequently browsed web pages while attempting to do homework assignments, which was a major source of distraction. ST5 (Female, learning in Thailand) openly admitted,

When you do not have any connection with your professors, you get tired of reading lecture materials. Then there’s no one around you to cheer you up or bring you back to the studying mood. I have more tendency to crush the deadline now than I was in high school.

The lack of connection with professors and classmates was the most mentioned problem because students could not see or interact with each other effectively in real time. ST19, a very outgoing and active student leader in high school, also actively sought help in university. Nevertheless, she found the circumstance challenging because “I could not look to peers or walk up to the front desk for support or any interactive measures to help us learn.” ST3 (Female, learning in China) illustrated her feeling of helplessness,

Being a high school student, you don’t know what university is going to be like. If you are in person, you could talk to a teacher, a tutor, or a supervisor. When you were online, you would have no idea whom to contact. Although there were many emails from the undergraduates’ office, you don’t know how to address them or who they are. I feel like entering a new world without any information and not really prepared.

Other cognitive problems, such as depression and anxiety, were intensified for students in online learning, as compared to hybrid learning. Students discussed how learning on Zoom did not feel natural and made it difficult to be engaged and present. Even when online support sessions were offered, “the reality is that people are not looking for online interaction anymore” (ST23, Female, learning in Hong Kong). More introverted students like ST2 (Female, learning in China) sadly confessed that learning and interacting on Zoom amplified their conditions of anxieties when speaking in public,

When I’m speaking to a screen, I get distracted. When I’m distracted, everything just goes to hell. I don’t remember anything. I underperform. And I just ramble about random stuff. And I don’t even know what I’m saying.

### 6.2. The hybrid-learning students: “A successful freshmen year”

**Figure 2:**
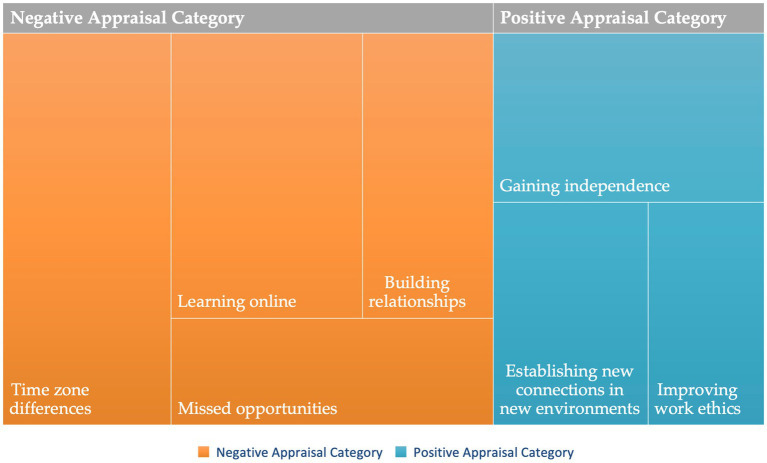
Appraisal codes visualization.

Hybrid-learning students faced issues and challenges common to their developmental stages, such as seeking independence, connections and belongingness, while contemplating the best options for their future career choices. It is critical to use students’ appraisal of the situation to understand their perspectives (Anderson et al., 2011). During analysis, student responses were coded into different appraisal themes under the main ‘positive’ and ‘negative’ codes (Figure 2). In response to assessing positive and negative dimensions and changes in college, students’ positive evaluations fell in three categories: *improving work ethics*, *establishing new connections in new environments*, and *gaining independence*, together with 37 sub-code coverages. The negative evaluations were regarding *learning online, missed opportunities, time zone differences,* and *building relationships*, with 67 sub-code coverages. All positive evaluations on independence and handling the basic life tasks were reported by students who moved to campus and had on-campus interactions.

Moving into a new environment contributed to young adults’ maturity and growth. Participants shared how they learned the basic tactics in a new country to “deal with the bank, health, healthcare, the paperwork” (ST 28, Male, in Canada). This process added to their sense of achievement as independent adults. ST22 (Male, in the United States) described his growth after arriving on campus,

I had to grow up very quickly in the past year living in college. A variety of things used to be things that my parents would take care of. Now I go to the doctor and get a bill with insurance on my own. But I’ve figured out that since I’m doing it now. It’s a big challenge. There’s a process to it. I think I have grown up much faster due to my situation at university.

Students viewed their first year of college as essential to building the foundation for academics and social development. Due to the pandemic restrictions, they constructed a limited on-campus support system, primarily with by roommates, classmates, and friendship groups as their microsystems. Peers were their important microsystem and source of support during difficult times. These students were overall pleased and satisfied with their first-year experiences: “I was still able to make friends and be social. I felt like it was a successful year because I was learning in my classes, and socially I formed good relationships” (ST24, Female, in the United Kingdom). ST21 (Female, in the United States) appreciated how peers played a role in supporting her in forming a new identity,

I was able to be around people that were kind of more like me and were interested in the same things as me… that helped sort of embrace my identity in a way. And yeah, I found it to be a really good year for growing.

Similarly, ST19 (Female, in the United Kingdom) stated, “I think it was good for me to move to this new country, even though my learning was online. I could access the facilities and know what my university looks like, rather than just seeing it on the screen.”

### 6.3. The fully online students: “All I wanted was to survive this semester”

The transition experiences were more negative for students studying abroad at home in Asia, in particular for those learning in a different time zone. Although appreciated the fact that they were being taken care of at home in a supportive and familiar environment, fully online students all faced physical and mental struggles. Issues and symptoms of worry, anxiety, and depression were associated with lack of sleep, lack of immediate institutional communication, and lack of regular social engagement.

ST7 (Female, learning in China) performed exceptionally well in an elite high school, but acknowledged frankly that she faced “a hardcore, difficult transitioning to university.” ST14 (Female, learning in Vietnam), who received an Australian world-class university’s merit scholarship, explained how the COVID-19 lockdown created a challenging environment to live and study,

Transitioning to an online university was extremely difficult because it was the time my country had a ban, so we could not go outside at all. The total isolation phase was four months, which was the entire duration of my university education online. The workload was extremely intense. I couldn’t relax. I couldn’t go outside. I was constantly worried about my country’s situation, my parents’ health, and my own health. I didn’t have social support from my friends or any relationships with teachers, that’s why I think it was even more difficult than normal [times].

ST5 (Female, learning in Thailand) stated, “the situation is so bad because you are always on a computer and your head just hurts all the time.” ST1 (Male, learning in China), a student who exuded confidence and optimism, quietly admitted: “I need a lot more assistance from friends and family to push me, [otherwise] I would be too tired and be in bed all day.” The participants in Thailand and China illuminated their struggles and confusion in more details.

I was struggling to keep up in class, and I probably did not sleep through one night for the past year. I took my classes throughout the night because all my classes were discussion-based, and I had to participate in them actively, so I stayed up. Sometimes I don’t sleep for two days in a row… I would give anything to someone who could just let me sleep, you know, for one night, like seven hours straight. All I wanted was to survive this semester. (ST2, Female, learning in China)

Well, it was very hard for me. A lot of people adjusted their times. I tried to sleep and wake up at 12 at midnight to take classed. Sometimes I woke up, but sometimes I didn’t. (ST7, Female, learning in China)

Some classes and quizzes would start at midnight, but some were in the mornings. I had to sleep, wake up, and then go back to sleep after taking a quiz. I slept at around 4 am and woke up at around 1 pm. Daylight savings got really confusing for me. I never slept like this late in high school. It’s been quite tiring. Yeah, I have been tired. (ST10, Female, learning in Thailand)

In contrast with the hybrid-learning students, fully online students remarked that their friend circle had *narrowed* during their first year in university. While they could communicate with more people on the phone or computer, they became less motivated. Many mentioned that they did not make any new friend online (ST13; ST15), and “I guess we were all burned out and did not care about socializing anymore” (ST17). Students needed to make trade-offs and decisions about whether and how to form relationships in a new environment: “building friendships with other kids – I had to actively give that up because I needed to get that sleep in that time” (ST2). “For me and a lot of people, the close friend circles became smaller, rather than broadened, which is a sad thing” (ST1). In Bronfenbrenner’s terminology, such experiences indicated that students’ microsystems, composed of the most immediate supporting relationships to their development, were not properly formed.

### 6.4. Perceived strategies and support

Students’ strategies fall into two categories: sticking to a strict schedule and reaching out to friends or mentors. All interviewees stressed the importance of finding a routine and maintaining a schedule to keep life on track during the chaotic time; “otherwise, many of my friends just break down” (ST1).

For interviewees studying at home in different time zones, it also meant trying to schedule designated times to be part of the local society for in-person interaction. Possible ways include connecting with friends in the same geographic area for day trips, shopping, partying, and meeting over coffee. This was an important strategy to “force yourself back into normal society and making you talk to more people” (ST3) so that students could feel connected with other human beings.

**Figure 3:**
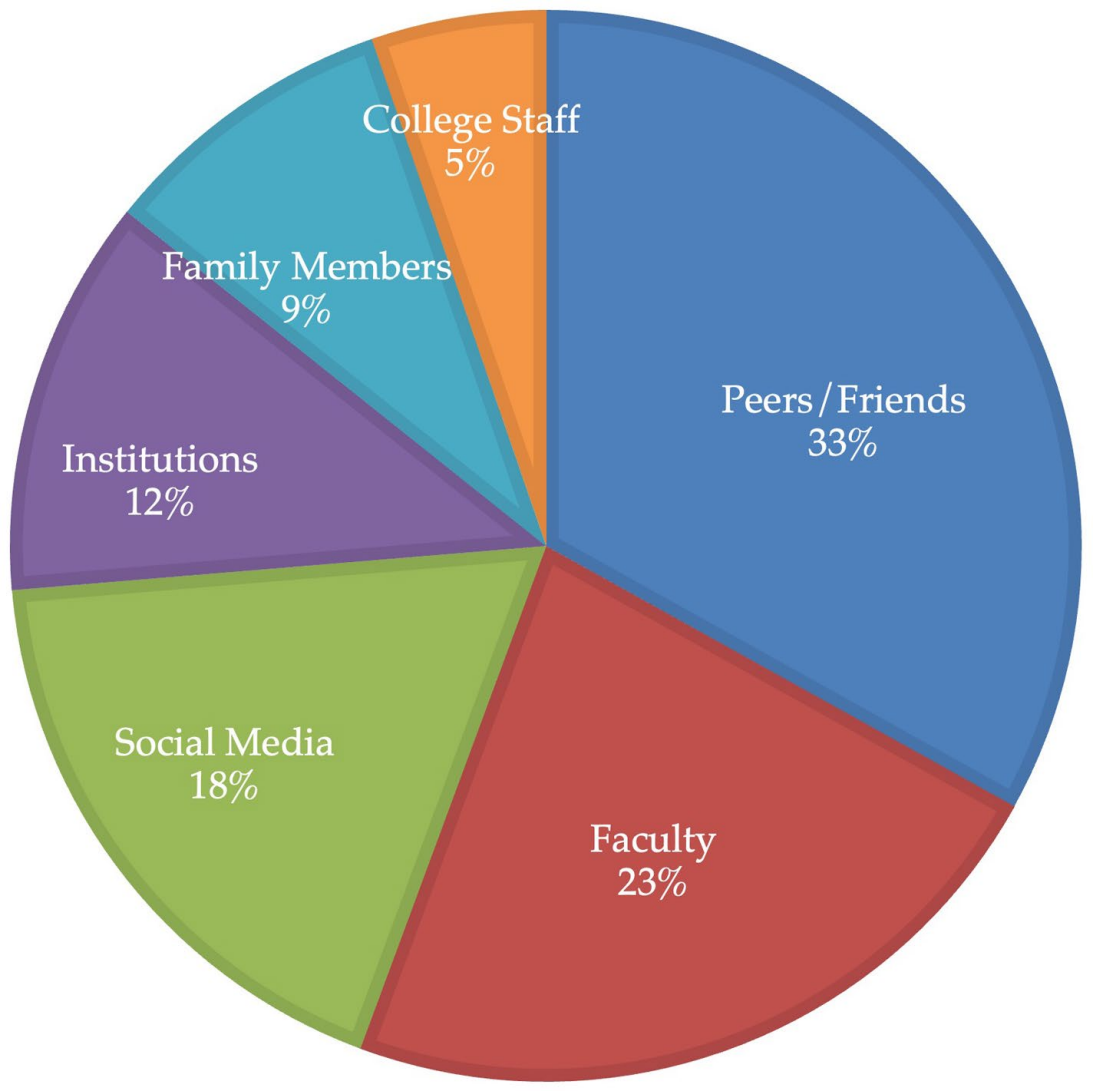
Support-system codes visualization.

In the survey, 27% of the student participants experienced monthly “lonely and isolated” feelings, 27% reported such feelings weekly, and 20% daily. The questions on constructing relationships were explored in-depth during the interviews. On the interview transcripts, the most frequently mentioned microsystem relationship codes were peers/friends (44 references), faculty (30 references), and social media (24 references). Less frequently mentioned codes were institutions (department) (16 references), family (12 references), and college staff (7 references; Figure 3).

As to where and how they established their microsystem support, it became evident that for students who were studying abroad at home, various social media platforms played an indispensable role in helping them to form social relationships and share information. Facebook class groups and group chats, Instagram posts and WhatsApp groups, and even Zoom messages became valuable channels for students to share their lives, reach out to friends, and establish new virtual friendships in universities. Friendship formation in their microsystems was key to their identity and social development.

## 7. Discussion

### 7.1. First-year international university students’ characteristics

*International student* is a broad definition, and we need to acknowledge the complexity of international student population (de Wit, 2008). In this study, the defined population are students who transitioned from international high schools in Asia to anglophone higher education institutions. This research thus faces a limitation in the small participant size, as the international high schools tend to be very small in scale. It has been consistently identified as an issue by scholars studying international schools and the International Baccalaureate population (e.g., Dickson et al., 2018; Jaafar et al., 2021). While the study purposefully sampled students of different nationalities and enrolled in different types of institutions in different host countries, circumstances would not be covered.

It remains unclear whether self-identifying as *Third Culture Kids* was conducive to student transitions, as predicted by early scholars (Schlossberg, 1981; Hayden, 2006; Pollock and Van Reken, 2006; Grimshaw and Sears, 2008). One potential reason behind this is in this research, *identity* was over-shadowed by other prominent influences, including pandemic-related environment changes, time differences, and place constraints. Whether and how being *Third Culture Kids* affects Asian students’ university transitions could be explored more in future research.

As suggested by literature, this specific group of international students seem to differ from the general international student pool. Due to their previous international school education, participants were highly proficient in the instruction language and did not experience the linguistic and cultural challenges identified by education and psychology researchers (Andrade, 2006; de Araujo, 2011; Smith and Khawaja, 2011; Ammigan and Jones, 2018). Many of them hold foreign passports in the host nations, thus are international students in the universities. This suggests that conducting large-scale surveys on international students and without accounting for their previous high school and living experiences may lead to misleading results and interpretations. The linguistic fluency and cultural familiarity could be the confounding factors influence students’ perceptions and evaluations of the circumstances. Many students perceived themselves as privileged, because they perceived facing fewer financial or digital strains than their under-resourced peers. In another words, the difficulties these participants encountered would be exacerbated for other international students studying at a distance.

### 7.2. “Multiple transitions” and “spatial–temporal displacement”

The findings from this research indicate a few important and interconnected theoretical issues in education and psychology. One is the multiple transition complications, and another is the space–time displacement. Although the transition theory has been widely applied in college student development, especially among the US veteran, transfer, and community college students, the international and cross-cultural dimensions received less attention (Tovar and Simon, 2006; Griffin and Gilbert, 2015; Ivins et al., 2017). Some researchers studied international students’ transitions; nonetheless, they focused explicitly on factors relating to English as the second language and cultural unfamiliarity (Heggins and Jackson, 2003; Zhang, 2016).

This study raises the issue concerning “multiple transitions.” It refers to the simultaneous and overlapping changes that these first-year university students experienced: (1) transition to university, (2) transition to a foreign education environment, and (3) transition to online learning amid a global pandemic. All were complex processes that entailed challenges and adjustments. As the theoretical transition model predicted, students’ relationships, routines, assumptions, and roles during the transnational university transition all altered to adapt to the new circumstances.

The first two transitions were anticipated changes, so some students might be able to utilize their psychological resources to prepare in advance (Anderson et al., 2011). However, the third transition was an unexpected change precipitated by the pandemic. Due to minimal in-person contact, these students perceived their “moving in” process to be unduly ongoing and prolonged, which exacerbated the transition complications. Being in different physical locations and scenarios influenced these interactions, routines, and responsibilities, as the microsystems students formed and the resources they amassed are significantly influenced by their locations. As a result, the relationships were not properly formed, and the routines are predicated to change once they enter university in person. It means they will undergo the “moving in” and “moving through” stages a second time when they move into university in person.

The place and time dislocation or displacement emerged as a critical factor influencing the participants, ultimately revealing the differentiated experiences between the 12 hybrid-learning and 18 fully online students. The place-related concept has captured the interests of environmental psychology and mobility researchers (e.g., Chow and Healey, 2008). Students were attached to the home and formed new place identities after entering university. This research shows that the ones studying *in situ* were limited in forming a new identity and developing social skills in a new cross-cultural environment.

Wang B. (2022) research on Chinese international graduate students studying at home for a shorter period also identified the distorted time zone issue. This study echoes the finding that studying abroad at home presents a peculiar condition. Students took classes and socialized in distorted time zones to compensate for place and opportunity losses. But first-year college students who are newly entering adulthood require more robust support for their development and psychological well-being (Nelson and Padilla-Walker, 2013; Katsumoto and Bowman, 2021). As a direct consequence, the individuals who took part in this research encountered a greater degree of cognitive and behavioral challenges and difficulties.

### 7.3. Sustainable practices to support quality education and student learning

The global higher education sector has tried to focus institutional efforts to incorporate the United Nations’ Sustainable Development Goals (SDGs), but the pandemic may lead to a shift in priorities during rapid digitalization and emergency remote teaching and learning practices (Crawford and Cifuentes-Faura, 2022). The Global Survey show that all higher education institutions were affected by COVID-19, and two-thirds replaced classroom teaching with distance teaching and learning (Marinoni et al., 2020). In such a context, many individuals, higher education institutions, and even international organizations believe that virtual mobility would present new international education opportunities (UNESCO, 2021). Nevertheless, the consequences and impacts of such a mode remain debatable, particularly across time zones and in the absence of in-person interaction.

While showing the challenges rising from time zone distortion, this study echoes recent quantitative empirical research on higher education students’ experiences worldwide. Studies have found that students could adapt to new learning and teaching practices, but the sense of isolation and loneliness negatively impacted their well-being, behavior, and learning (Cifuentes-Faura et al., 2021). In confined environments at home, students faced difficulties following the course online, spent more hours studying, and achieved lower academic performance (Faura-Martínez et al., 2022). The described hurdles and difficulties may explain why studying abroad *in situ* was not optimal and 79% of students, according to a global survey, desired to study abroad on campus (Nuthall, 2021).

On the other hand, this study highlights how changes in a student’s environment could either facilitate or impede their transitions and well-being, which offer implications for the future of online education in a sustainable manner.

#### 7.3.1. Exosystem-level support

There appears to be a disconnect between student services offered by institutions and those acquired by student participants during the pandemic. Not receiving adequate social support and security protection from others and instructors in times of need has presented hardship to domestic university students worldwide, including Cambodia, Nigeria, Oman, and Spain (Cifuentes-Faura et al., 2021). Similarly, in this study, no international student responded positively when asked if the institution or any organization in their exosystems has been helpful. Picton and Kahu (2021) have proposed that students be taught proactive skills to actively seek support services (academic advising, career advice, and well-being services). Other research also indicates that colleges may employ multiple offices and faculty-student interactions to promote student development and online learning (Baik et al., 2019; Trolian et al., 2020; Borowiec et al., 2021; Kim et al., 2021).

Nonetheless, for the participants with the awareness and skills to seek help, they perceived limited assistance at a distance. Students stated that if faculty members seemed busy, it was an indication that they did not wish to be contacted. For first-year international students, arranging an online appointment with the student offices was challenging due to time differences. ST7 (Female, in China), enrolled in a small selective liberal arts college in the United States, explained the situations at her institution,

Most of my classes were synchronous; they expected participation and you to be online on Zoom. My friend talked to the college about how hard it was for the Chinese students to be online around 3 am and 4 am. But they were like, “Oh, well, you guys are the minority of our school.” Because we were a small college, it was hard for them to take care of everyone.

At the same time, there are higher educational institutions that offered sustainable and effective practices for their students. Providing dedicated time and places for these international students studying in different time zones have proved to be a supportive strategy. For instance, Franklin Marshall College and New York University were exceptional in catering to the needs of international students studying abroad at home (Moja, 2021; Mann and Mennicke, 2022). The latter allowed their students to “Go local” and choose one of the global branch campuses in New York City, Abu Dhabi, or Shanghai. These approaches created an environment conducive to meeting students’ academic and social demands and enhancing their well-being. Moving forward, practices and models like these would benefit higher education institutions wishing to develop and expand their hybrid and online programs to international or distance students.

#### 7.3.2. Microsystem-level support

During the epidemic, microsystems, even virtual ones, are essential for supporting first-year students. It is even more crucial and apparent when social distance is required, and only virtual friendships are available. Students’ responses on friends being the most helpful source of support during the transition corroborated findings on the sense of belonging and formation of friendships in higher education (van Gijn-Grosvenor and Huisman, 2020; De Sisto et al., 2021). The construction of virtual microsystems *via* digital platforms lends credence to the notion that social media could positively contribute to undergraduate students’ international educational experiences (Chang et al., 2021; Nguyen et al., 2021). Aside from academic learning, higher education institutions could utilize various digital platforms to serve as bridges for students to build communities and connect with each other within the same locations and time zones.

## 8. Conclusion

The pandemic presented a unique opportunity to understand how students transitioned from high school to university abroad and in what ways they were affected by the environment. This research finds that firstly, first-year international students did not evaluate online learning as very effective due to a lack of engagement and an increase in distraction. Secondly, there was a remarkable contrast in the experiences and well-being of fully online and hybrid-learning students. At a macro-environment level, the pandemics led to government lockdowns, border controls, and travel restrictions. Following these strict measures, the complex “multiple transitions” and “time and place displacement” disrupted international students’ establishment of and interaction with various components from microsystems to exosystems. As such, higher education institutions should target practices and services to support international students in complex transitions and difficult situations. This study contributes to empirical knowledge of transnational transitions and theoretical development on transitions during cross-cultural, temporal, and spatial environments.

This group of international first-year students were relatively well-prepared for universities with prior cross-cultural experiences. They did not confront the same linguistic and financial obstacles that other international and disadvantaged students did. Nevertheless, the results show that time zone distortion, place dislocations, missed opportunities, and narrowed support networks were detrimental to student growth and well-being at a crucial developmental stage. In the post-pandemic world, hybrid and distance learning will continue to be an important feature in all forms of education, as will students’ transitions into international higher education. The experiences and resources acquired from online teaching and learning hold practical value for the future of sustainable higher education practices, including retaining traditional in-person instruction features and building online competencies (Turnbull et al., 2021). It would be fruitful to continue research to investigate effective and sustainable institutional practices, especially catering to the conditions of students at a distance and in different time zones.

## Data availability statement

The interview audio and transcript data are not readily available as per ethics requirements. Requests to access the datasets should be directed to the author LW, wangliz@bc.edu.

## Ethics statement

The studies involving human participants were reviewed and approved by Boston College Institutional Review Board. The participants provided their written informed consent to participate in this study.

## Author contributions

The author confirms being the sole contributor of this work and has approved it for publication.

## Funding

This study is supported by the Council of International Schools and Boston College’s Center for International Higher Education.

## Conflict of interest

The author declares that the research was conducted without any commercial or financial relationships that could be construed as a potential conflict of interest.

## Publisher’s note

All claims expressed in this article are solely those of the authors and do not necessarily represent those of their affiliated organizations, or those of the publisher, the editors and the reviewers. Any product that may be evaluated in this article, or claim that may be made by its manufacturer, is not guaranteed or endorsed by the publisher.

## Footnotes

^1^Source: compiled and adapted by the author from Anderson et al. (2011, p. 39) and Patton et al. (2016, p. 49).

^2^Because international schools are very small in size, school and interviewee information is presented as broadly and vaguely as possible to ensure confidentiality.
